# The effect of functional mandibular advancement for adolescent patients with skeletal class II malocclusion on the TMJ: a systematic review and meta-analysis

**DOI:** 10.1186/s12903-022-02075-8

**Published:** 2022-03-03

**Authors:** Lan Ding, Rui Chen, Jiaxin Liu, Yuan Wang, Qian Chang, Liling Ren

**Affiliations:** Department of Orthodontics, School of Stomology, Lanzhou University, No.199, Donggang West Road, Lanzhou City, 730000 Gansu Province China

**Keywords:** Functional mandibular advancement, Temporomandibular joint disorders, Class II malocclusion, Orthodontics, Systematic review

## Abstract

**Objectives:**

This study aimed to assess whether functional mandibular advancement (FMA) will cause temporomandibular joint disorders (TMD) or have side effects on temporomandibular joint (TMJ) in adolescent patients.

**Methods:**

All searched databases, including PubMed, Web of Science, EMBASE, Cochrane Central Register of Controlled Trails and Scopus were searched. Gray literature and unpublished literature was also searched. Randomized controlled trails (RCT) and non-randomized studies of the effects of interventions (NRSI) directly observe the condition of adolescent patients’ TMJ after finishing treatment will be considered to include in our study. According to Cochrane Handbook, Cochrane Collaboration risk of bias tool was used to assess the quality of included RCTs, and Risk of Bias In Non-randomized Studies of Interventions (ROBINS-I) tool was used to assess the quality of included NRSIs.

**Result:**

Finally 18 researches were evaluated as eligible to include in this study. 5 of the studies were RCTs, 8 were NRSIs and 5 were systematic reviews. The data of RCTs and NRSIs were statistically pooled in meta-analysis. The number of samples under investigated among primary studies was 579 individuals,there were 80 patients who developed temporomandibular symptoms during or after treatment. But all the subjective symptoms disappeared during follow-up time. The statistical outcomes proved that patients received FMA didn’t show more tendency to develop temporomandibular symptoms [I^2^ = 27%, OR = 0.54, 95%CI (0.33,0.87), p = 0.01].

**Conclusion:**

(1) TMJ symptoms may occur during the functional oral appliance wearing, but the symptoms will release or disappear after treatment or during the follow-up period. (2) Less convincing evidence indicates that slightly previous TMD and condyle-glenoid fossa relationship will be improved after treatment. (3) There is TMJ disc anterior displacement observed during treatment, but most of them will return to the normal position later. (4) Moderate evidence support that FMA will not have side effects on TMJ of adolescent patients.

**Supplementary Information:**

The online version contains supplementary material available at 10.1186/s12903-022-02075-8.

## Introduction

Skeletal class II malocclusion is regarded as a common disease among people [1–4], and its mechanism is either the overgrowing of maxillary, or the deficient of the mandibular [5]. For the second type, functional mandibular advancement (FMA) has become an effective treatment for skeletal class II malocclusion [6–8], and it is commonly used in adolescence who still have growing potential [9], usually cervical vertebral maturation stages (CVMS) I–IV [10], the age may range from 8 to 16 years, by having mandibular forward positioning to stimulate mandibular growing [11].

TMD is a disease that occurring in TMJ area and adjacent soft tissues, including muscle or TMJ pain to palpation, TMJ clicking, and the dysfunction of mandibular movement [12]. Often 40%-75% of the populations have at least one of TMD symptoms [13]. The reasons that caused TMD is complicated, and the factors consistently associated with TMD included other pain conditions (e.g. chronic headache) [14], sleep apnea, mental condition (e.g. anxiety, depression) [15], trauma [16, 17] and so on. Occlusion factors might be one of the main causes related to TMD [18]. TMD is categorized as intra-articular derangement (within the joint) and extra-articular derangement (involving the surrounding musculature) [17]. Articular disk displacement involving the disc-condylar relationship is mostly common seeing in intra-articular dysfunction. Forward mandibular positioning will make the disc at a more advanced position, which might change the disc-condylar relationship and induce TMD. The impact of functional mandibular advancement on signs of temporomandibular dysfunction observed in previous published researches including TMJ capsular pain to palpation, TMJ sound, muscle pain to palpation [6, 19]. And cone beam computed tomography (CBCT) is also widely used to help diagnosing TMJ dysfunction [12, 20, 21] by evaluating the morphology of condylar and whether the condylar position remains normally.

Although many researches in animals have proved there was no TMJ symptoms after functional mandibular advancement [7, 22], controversy still exists because of the adaption mechanism of the TMJ during functional mandibular advancement [23, 24]. Some researchers hold the opinion that functional mandibular advancement will have positive impact on TMJ reconstruction, which can help modifying better condyle-glenoid fossa relationship [25]. But on the contrary, others think that functional mandibular advancement might cause TMDs, since it breaks the balance of occlusal relationship [26–28]. However, the researches above were all based on clinical trials; the sample was not enough to offer convinced evidence, also, the potential bias from researchers selecting cases may affect their results. So, it seems that there was no high-quality evidence of existing literature regarding the impact of functional mandibular advancement on TMJ.

Some previous systematic reviews and meta-analyses published by Kurt Popowich [29] and Laura Ivorra-Carbonell [30] analyzed the morphology of TMJs after functional mandibular advancement, both of their researches found that the condyle was at a more advanced position, with the remodeling of the condyle and glenoid fossa; and no significant adverse effect was detected. But the results were inconclusive. Their evaluation of this problem was qualitative, which made the results less convinced. This systematic review and meta-analysis was undertaken to answer whether functional mandibular advancement would have adverse effects on TMJs in adolescence with quantitative measurement since no quantitative assessment was undertaken in previous researches.

## Objectives

This systematic review and meta-analysis aims to evaluate whether functional mandibular advancement will cause temporomandibular joint disorders or have side effects on temporomandibular joint in adolescent patients.

## Materials and methods

### Protocol and registration

The protocol of this systematic review was developed and registered prospectively in PROSPERO (www.crd.york.ac.uk/prospero, CRD42020157906). This review was performed following the Cochrane Handbook for Systematic Reviews of Interventions [31]. The MOOSE guideline [32] and PRISMA statement [33] were followed by all of the authors to report the results.

### Eligibility criteria

The following selection criteria were applied for this study.

1. Study design: randomized and controlled clinical trials, non-randomized studies of the effects of interventions, along with systematic review and meta-analysis, which considered the TMJ condition of patients after functional mandibular advancement with a period for observation.
2. Participants: adolescent patients who had received functional mandibular advancement.
3. Interventions: functional mandibular advancement appliance was used to improve profile and class II malocclusion.
4. Inclusion and exclusion criteria: Inclusion criteria: a. All controlled trials about the influence on TMJ caused by functional mandibular advancement; b. Patients with good compliance till the end of treatment are of over 80% of the case number; c. Patients who didn’t receive orthodontic treatment, orthognathic surgery, or TMD treatment before; d. The patients’ age should be under 16 years; e. The studies are considered to be high quality or medium quality. Exclusion Criteria: a. Repeated researches; b. Studies that didn’t have control trials or before and after comparison; c. The patients whose age is over 16; d. The studies are considered to be of low quality by the criteria ordered by Cochrane Handbook for systematic reviews of interventions.
5. Outcome measures: we set 5 main indicators to assess the outcomes; they were the TMJ morphology before and after treatment, reported muscle disorders, position of TMJ disc before and after treatment, reported TMJ noises, reported Oral-facial pain and reported TMJ pain.

### Information sources, search strategy, and study selection

International databases were used to find published articles from the opening of the database to August 2021. All searched databases, including PubMed, Web of Science, EMBASE, Cochrane Central Register of Controlled Trails and Scopus were thoroughly searched using keywords: mandibular advancement, mandibular forward positioning, functional mandibular advancement, Herbst appliance, activator appliance, bionator appliance, twin-block appliance, Fränkel appliance, Forsus appliance, temporomandibular joint, temporomandibular joint disease, craniofacial pain, condylar resorption, class II malocclusion, orthodontics, randomized clinical trial, controlled clinical trial, placebo, double-blinded method and single-blinded method (searching strategies are supported in Additional file 1). Also, gray literature was sought by hand search, and contacting the author to ask for the original text of meetings and conference abstract when needed.

Search evaluation and the assessment of risk of bias were made by 2 researches independently, and if there existed any dispute, the evaluation should be reevaluated by the third researcher. Original articles, case reports, case series, meetings and conference abstracts which are published in English are considered in this study. We also search for bibliographic survey to enhance the sensitivity and to select more articles. Unpublished literature was searched electronically in ClinicalTrials.gov (www.clinicaltrials.gov) and the National Research Register (www.controlled-trials.com).

Full text or brief of all studies, reports, meeting or conference abstracts resulted from advanced search were extracted. After detailed screening the topic, abstract, and the full text, and removing the duplicates, so that the unrelated studies could be excluded and the related ones could be selected. The articles considered for this study including randomized controlled trails, non-randomized studies of the effects of interventions, and systematic reviews (the PRISMA flow diagram was reported in Additional file 2).

### Data items and collection

For each RCT and NRSI, data were extracted based on topic, published year, type of study, total sample size, the ratio of gender, average age of patients, total amount of advancement, follow-up time, examination after finishing the treatment, outcome and result synthesis. All the data were performed in Table 1.

**Table 1:** Characteristics of all included controlled trails

Study ID (country)	Type of study	Sample size	Gender (M/F)	Average age (year)	Total amount of advancement	Treatment period	Follow-up time	Examination on TMJ	Outcome	**Result synthesis**
A. A. Franco (2002) Brazil	RCT	84	43/41	10.3y	< 6 mm	18 m	18 m	TMJ examination + Qusetionare + MRI	ac	The findings showed a low prevalence (3.57%) of disc displacement related to functional mandibular advancement in the 112 temporomandibular joints
Aidar et al. 2009 Brazil	NRSI	32	16/16	12.8y	< 6 mm	12 m	12 m	TMJ examination + Qusetionare	a	In 42 (65.62%) of the 64 TMJs, after follow-up, the disc had returned to normal limits. In 22 TMJs (34.37%), no changes were observed after follow-up
D.D. Güner (2003) Turkey	RCT	17	9/8	12.8y	> 6 mm	6 m	6 m	CBCT + TMJ examination	ac	The results indicate that new bone formation in the mandibular condyles seems to contribute to the increase in mandibular prognathism resulting from functional jaw orthopaedics
Gabriela Modesti-Vedolin et al. (2018) Brazil	NRSI	18	10/8	8.4y	< 6 mm	2 m	2 m	TMJ examination + CBCT	b	82.6% to 88.9% of the patients didn’t report the discomfort of TMJs, and no disc displacement was observed
Gero Kinzinger et al. (2006) Germany	NRSI	15	8/7	15y	> 6 mm	5 m	7.5 m	TMJ examination + MRI	c	Comparison of baseline and post-treatment findings revealed that none of the joints exhibited a treatment-induced deterioration in the disc-condyle relationship, while the relationship improved in five joints
Hans Pancherz et al. (1998) Germany [34]	NRSI	20	10/10	12y	< 6 mm	7.4 m	5y	Questionaire + TMJ examination + MRI	cde	When summarizing the anamnestic, clinical and magnetic resonance imaging findings five subjects (25%) exhibited moderate to severe signs of temporomandibular disorders ranging from partial to total disk displacement or “deviation in form” of the condyle. Another three subjects (15%) showed mild symptoms of temporomandibular disorders with either small condylar displacement or subclinical soft tissue lesion
HY Elfeky (2018) Egypt	RCT	40	0/40	12.5y	< 6 mm	9.4 m	6 m	CBCT + TMJ examination	a	Results of the net effect of the Twin Block on the osseous TMJ components and joint spaces showed a significant change in the condylar dimensions and significant forward positioning of the right and left condyle
Ken Hansen et al. (1990) Germany	NRSI	38	19/19	12.4y	< 6 mm	6 m	7.5y	Questionaire + TMJ examination + CBCT	df	No tenderness or lateral or posterior palpation of the TMJ was recorded in any of the subjects
Niko C. Bock et al. (2018) Germany	NRSI	72	32/40	13.6y	> 6 mm	1.8y	18.3y	TMJ examination	bdf	79–91% of the patients were free of TMD signs and symptoms (RDC/TMD and DC/TMD). The TMD prevalence fluctuated: 21% at the beginning of treatment, 9% after treatment, 15% after follow-up
Sabine Ruf et al. (2000) Germany	NRSI	62	27/35	14.4y	> 6 mm	7.2 m	1y	MRI + TMJ examination	def	Over the entire observation period from before treatment to 1 year after treatment, bite-jumping with the Herbst appliance: (1) did not result in any muscular TMD; (2) reduced the prevalence of capsulitis and structural condylar bony changes; (3) did not induce disc displacement in subjects with a physiologic pretreatment disc position; (4) resulted in a stable repositioning of the disc in subjects with a pretreatment partial disc displacement with reduction; and (5) could not recapture the disc in subjects with a pretreatment total disc displacement with or without reduction
Hans Pancherz et al. (2015) Germany	NRSI	28	24/4	13.4y	< 6 mm	1y	31.8y	CBCT + TMJ examination	bcd	At the 32-year follow-up, six patients had TMJ clicking and one patient had TMJ pain
GSM Kinzinger et al. (2006) Germany	NRSI	20	11/9	16y	< 6 mm	7.3 m	3.7y	MRI + TMJ examination	abd	Upon adoption of the therapeutic position, the condyles were displaced from the centric position within the fossa toward caudal and ventral. At the end of treatment, they returned to their original position
Fangfang Gong et al. (2011) China	NRSI	22	8/14	11.7y	< 6 mm	9.3 m	None	CBCT	bcf	The mandibular condylar growth was directed Superiorly (2.7 mm) and posteriorly (3.6 mm) (P < 0.01), the glenoid fossa was displaced in a inferior (1.5 mm) (P < 0.01) and posterior (0.8 mm)(P < 0.05) direction, the effective TMJ changes showed a pattern similar to condylar growth in a superior (4.2 mm) and posterior (2.7 mm) direction(P < 0.01), the mandibular rotation was slightly clockwise(P > 0.05)
Stephen D. Keeling (1995) USA	RCT	131	52/79	9.5y	> 6 mm	6 m	6 m	TMJ examination	bdef	Subjects with a TMJ sound, joint pain, and/or muscle pain at follow-up were more likely those who had the sign at baseline (P < .01). Early treatment with bionators did not place healthy children without these signs at risk for developing these signs
Weiwei Chen (2016) China	RCT	15/15	9/6	12.4y	< 6 mm	7 m	2y	CBCT	ac	CBCT showed a crescent-shaped hyperplasia at the posterior-superior border of the condyles that the outer edge was highly dense and the inner region was lowly dense in 14 patients
Yuan-yuan Jiang (2020) China	RCT	26	13/13	10.8y	> 6 mm	6 m	8 m	CBCT	ac	Twin-block group exhibited more obvious condyle-fossa modifications and joint positional changes than control group

Table 1 basic characteristics of the included studies.

Outcome: a. TMJ morphology b. Muscle disorders c. Position of TMJ disc d. TMJ clicking e.Oral-facial pain f. TMJ pain to palpation.

### Risk of bias/quality assessment in individual studies

After selection of the studies in terms of the topic, abstract and design of the experiment, to assess the quality of the selected studies, Cochrane Collaboration risk of bias tool [35] was used to assess the quality of included RCTs, and seven criteria were analyzed to grade the risk of bias inherent in each study, including random sequence generation, allocation concealment, blinding of participants and personnel, blinding of outcome assessment, incomplete outcome data, selective reporting and other potential source of bias. Studies with at least 1 criterion of high risk would be regarded as having a high risk of bias overall, and excluded from meta-analysis. Risk of Bias in Non-randomized Studies of the Effects of Interventions (ROBINS-I) tool was used to assess the quality of included non-randomized trials, also seven criteria were used to evaluate the inherent risk of bias in each study, including confounding, selection bias, classification of interventions, deviation from interventions, missing data, measurement of outcome, and selection of reported result. Studies with at least 1 criterion of high risk would be regarded as having a high risk of bias overall, and excluded from meta-analysis. According to the guide of Cochrane Handbook, systematic reviews are considered as high quality evidence and capable of including in systematic review.

### Summary measures and approach to synthesis

Heterogeneity of the included studies was gauged by assessing the treatment protocol—follow-up year, gender ratio, publish year, treatment period and total amount of advancement. Statistical heterogeneity was assessed by a forest plot in conjunction with 95% confidence intervals. P value below 0.1 meant significant heterogeneity. Index among studies was determined, applying Z test and I-squared, according to heterogeneity results, random or fixed model was used for estimation. The results for developing TMD were expressed as odds ratios (OR).

### Risk of bias across studies

Funnel plot was drawn to identify and evaluate publication bias.

### Additional analyses

Sensitivity analysis was pooled to deal with studies with higher risk of bias. Publication bias, and other potential sources of heterogeneity including follow-up time, gender ratio, treatment period and total amount of advancement in included studies would be assessed by subgroup analyses. Meta-analysis, sensitivity analysis and subgroup analyses were undertaken using Review Manager Ver 5.3 software.

## Result

### Study selection and characteristics

A sensitive search including 1015 articles was found out. After reading the topic and abstract, 25 studies were selected in this study. And according to the exclusion criteria, 4 articles were excluded, so that 21 articles were included in this study finally. The flow of study selection is performed in PRISMA flow diagram (Additional file 2).

PRISMA flow diagram of article retrieval.

### Risk of bias within studies

**Fig. 1:**
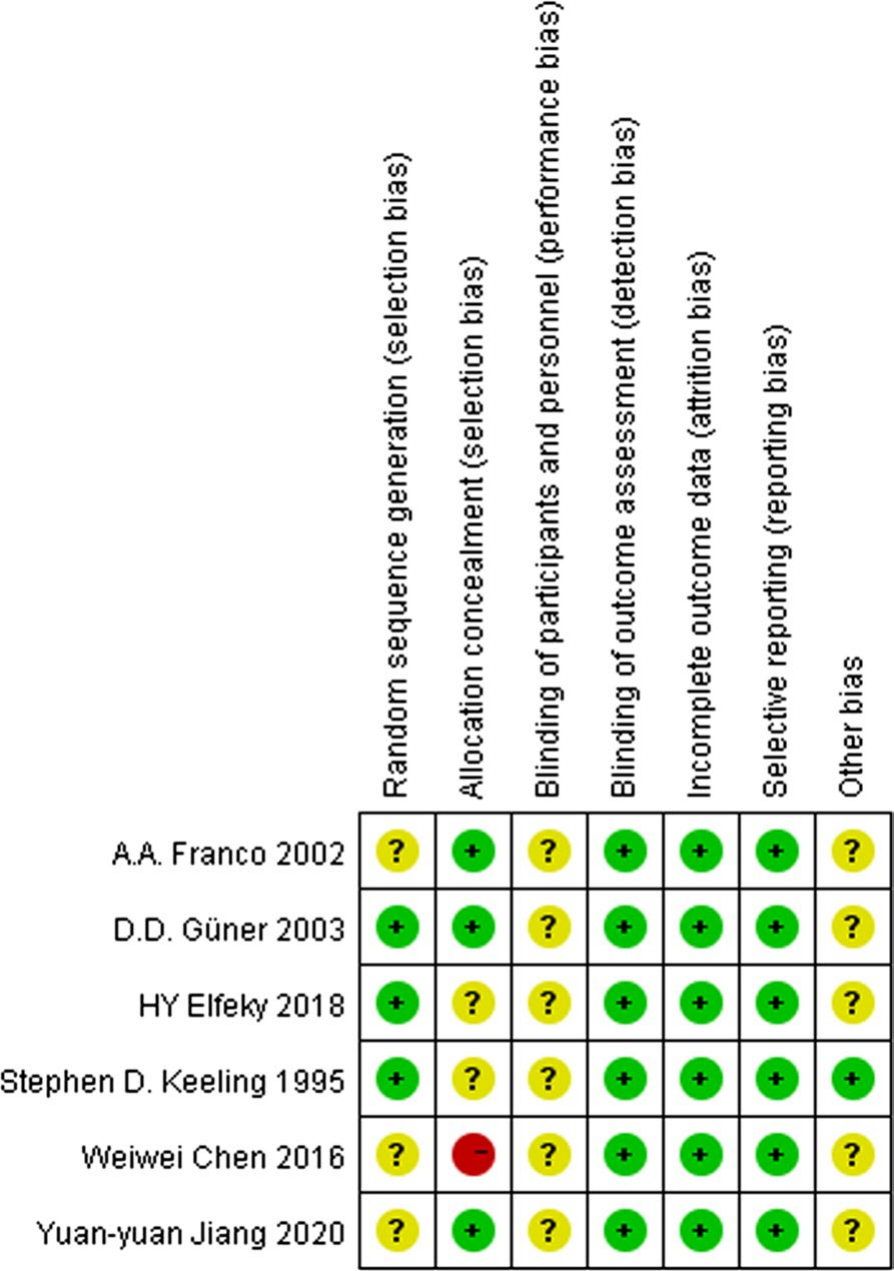
Risk of bias summary

**Fig. 2:**
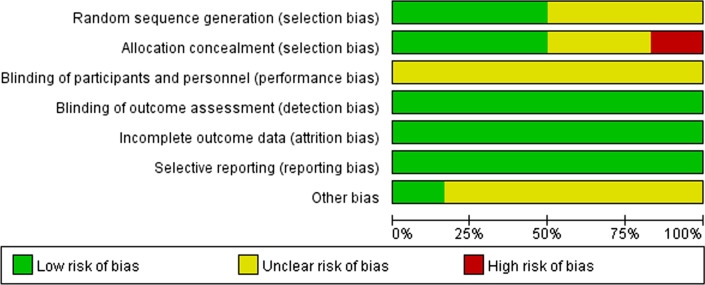
Risk of bias. Review author’s judgments about each risk of bias item presented as percentage across all included studies

Seven criteria were pooled to analyze the inherent risk of bias of each included RCTs, including random sequence generation, allocation concealment, blinding of participants and personnel, blinding of outcome assessment, incomplete outcome data, selective reporting, and other potential sources of bias. The result of quality assessment was showed in Fig. 1. And an overview of author’s judgments concerning all aspects of risk of bias is presented in Fig. 2. Also ROBINS-I tool was used to assess the quality of included NRSIs, seven criteria including confounding, selection bias, classification of interventions, deviation from interventions, missing data, measurement of outcome, and selection of reported result were used to analyze the risk of bias. The result was showed in Table 2. Additionally, all included studies declared that there was no selective reporting of results in their studies. Considering all the patients should be informed the orthodontic treatment plan, so we couldn’t include any double-blinded methods. All authors stated in the literature that there was no attempt to blind the participants, but all the patients were not informed of others’ treatment plan. In particular, our included articles mentioned blinding of all assessors.

**Table 2:** Bias assessment using the ROBINS-1 tool

ROBINS-1: risk of bias in non-randomized studies of the effects of interventions							
	Confounding	Selection bias	Classification of interventions	Deviation from interventions	Missing data	Measurement of outcome	Selection of reported result
Aidar et al. (2009)	Low risk	Moderate risk	Moderate risk	Low risk	Low risk	Low risk	Low risk
Gabriela Modesti-Vedolin et al.	Low risk	Moderate risk	Low risk	Moderate risk	Low risk	Low risk	Low risk
Gero Kinzinger et al.	Low risk	Low risk	Moderate risk	Low risk	Moderate risk	Low risk	Low risk
Hans Pancherz et al. (1998)	Moderate risk	Low risk	Moderate risk	Moderate risk	Low risk	Low risk	Low risk
Ken Hansen et al. (1990)	Moderate risk	Low risk	Moderate risk	Low risk	Low risk	Low risk	Low risk
Niko C. Bock et al. (2018)	Moderate risk	Low risk	Low risk	Low risk	Low risk	Low risk	Moderate risk
Sabine Ruf et al. (2000)	Low risk	Moderate risk	Moderate risk	Low risk	Low risk	Low risk	Low risk
Hans Pancherz et al. (2015)	Low risk	Low risk	Moderate risk	Low risk	Low risk	Low risk	Moderate risk
GSM Kinzinger et al. (2006)	High risk	Moderare risk	High risk	Low risk	Low risk	Low risk	Low risk
Fangfang Gong et al. (2011)	High risk	High risk	Moderate risk	Low risk	Low risk	Low risk	Moderate risk

Therefore, overall, 2 NRSIs and 1 RCT were deemed as low quality and they were not eligible for meta-analysis, other 13 studies were eligible to be pooled in quantitative assessment. According to Cochrane Handbook, systematic review is considered to offer strong evidence, so 5 systematic reviews were also included, and the conclusion would be stated in discussion of this study.

### Results of individual studies, meta-analysis, and additional analyses

#### Results of meta-analysis

**Fig. 3:**
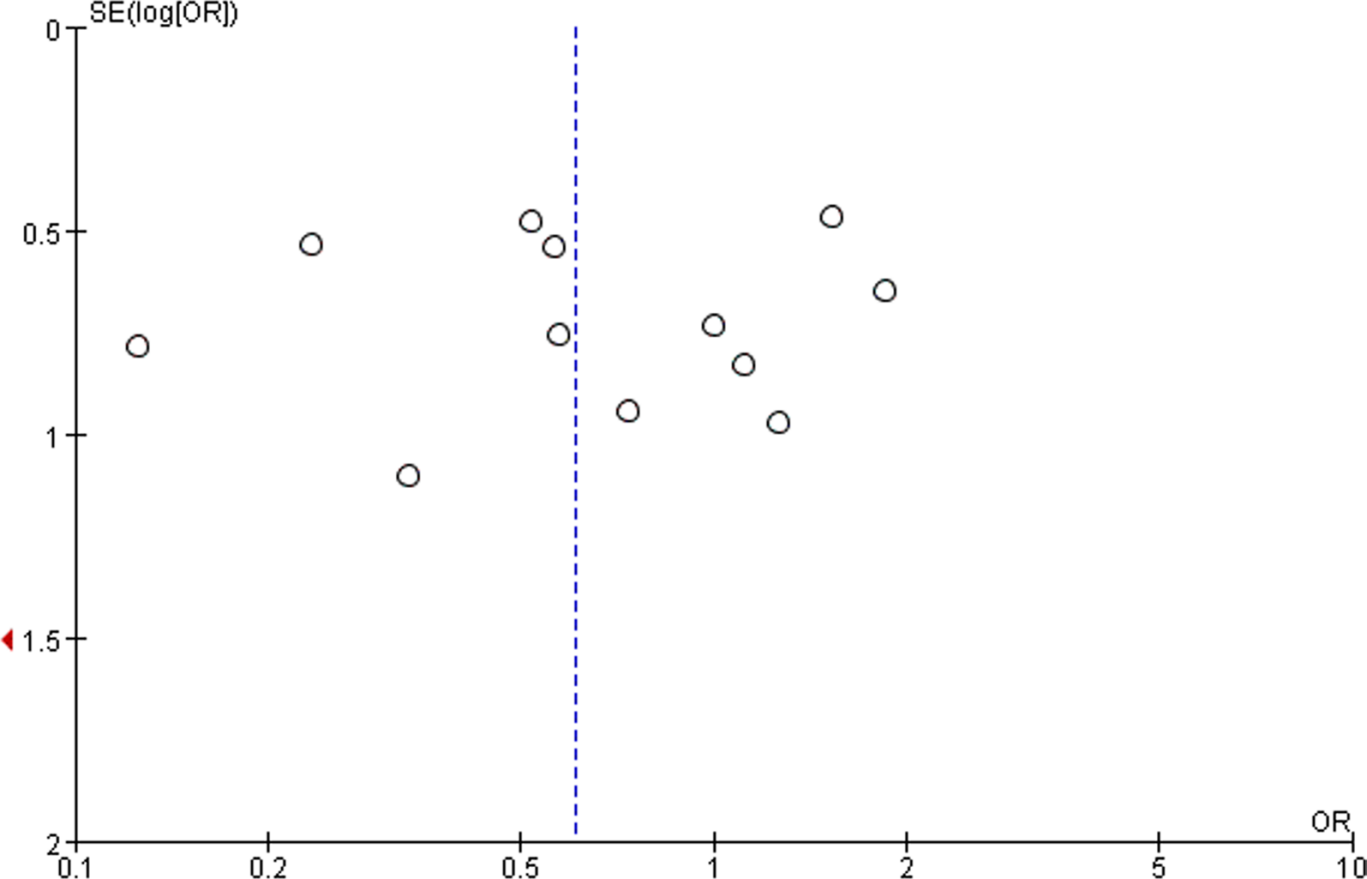
Funnel plot to display the publication bias

Review Manager Ver5.3 software was utilized to analyze data. Sensitivity analysis was also undertaken to determine effective studies in terms of heterogeneity. Funnel plot was performed to assess publication bias (Fig. 3), and the results showed that there was little publish bias among studies.

**Fig. 4:**
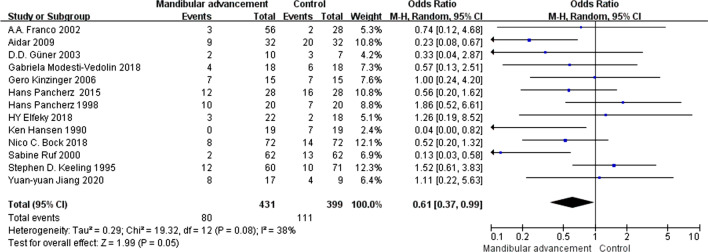
The comparison between pre and post-treatment (OR)

In this meta-analysis, we used OR as a statistical indicator, and the result showed in Fig. 4 that the degree of heterogeneity is significant [I^2^ = 38%, OR = 0.61, 95%CI (0.37, 0.99), P = 0.05]. So that the random effects model was used to analyze the data. Meta-analysis performed that compared with pre-treatment, patients who received the intervention of functional mandibular advancement didn’t appear to have more serious TMD or attain new TMJ symptoms, and the consequence had statistical significance.

**Fig. 5:**
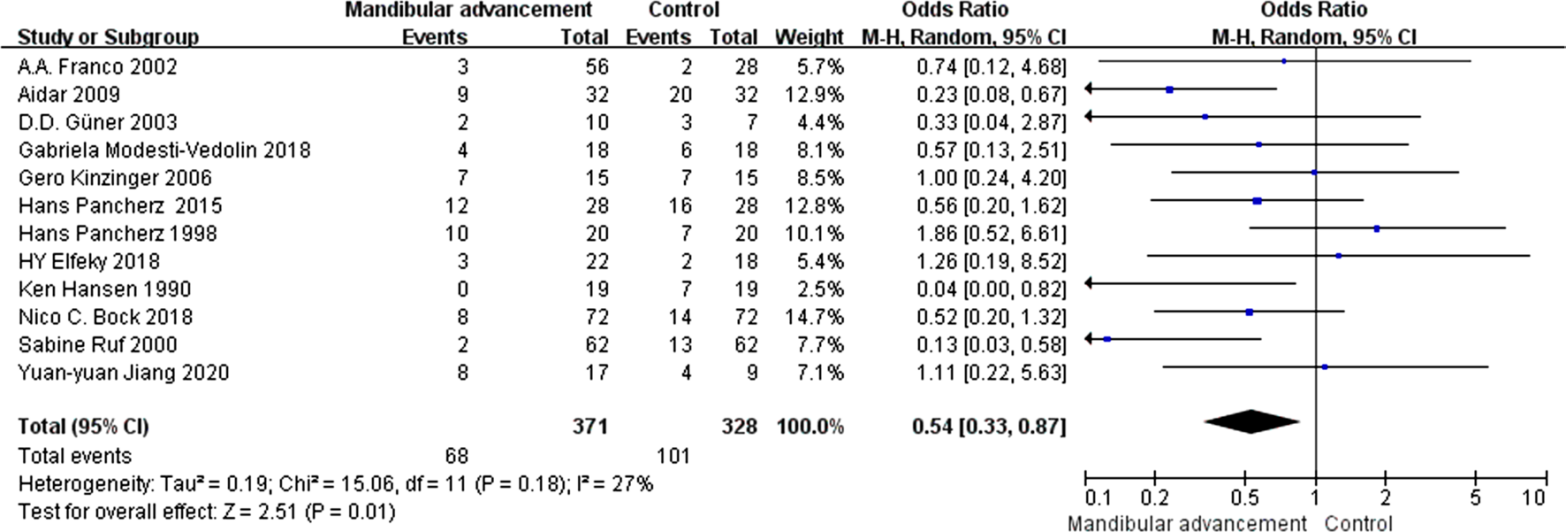
The result of sensitivity analysis (after removing the study of Stephen D. Keeling)

Sensitivity analysis to examine role of each primary study in heterogeneity showed that research done by Stephen D. Keeling had the most effect on heterogeneity, and heterogeneity decreased evidently after removing this study [I^2^ = 27%, OR = 0.54, 95%CI (0.33, 0.87), p = 0.01]. The result was performed in Fig. 5.

### Results of included systematic reviews

After electronic searching, finally 5 systematic reviews were included in this study because they were directly related to the review topic and met the selection criteria. The synthesis of results and conclusions was demonstrated in Table 3. And the results of these studies would be assessed in the following 3 aspects.

**Table 3:** The synthesis of results and conclusions of included systematic reviews

Study ID	Country	Results/conclusion
Kurt Popowich et al. (2002)	Canada	The MRI studies did not provide conclusive evidence of osseous remodeling or condyle position change. The tomography study demonstrated minor condyle position change. Methodological deficiencies prevented major conclusions regarding disc position
Laura Ivorra-Carbonell et al. (2016)	Spain	After treatment with functional appliances, the condyle was found to be in a more advanced position, with remodeling of the condyle and adaptation of the morphology of the glenoid fossa. No significant adverse effects on the TMJ were observed in healthy patients and the appliances could improve joints that initially presented forward dislocation of the disk
Lucas Garcia Santana et al. (2020)	Brazil	Low to very low certainty of evidence indicated that incremental mandibular advancement resulted in greater gains in mandibular length (MD = 0.89 [0.38, 1.34],p = 0.0005), anterior mandibular displacement (MD = 0.73 [0.40, 1.06], p < 0.001) and SNB angle (MD = 0.44 [0.02, 0.85],p < 0.04)
Karma Shiba Kyburz et al. (2019)	Switzerland	Currently existing evidence from controlled clinical studies on humans indicates that functional appliance treatment is associated with positional and skeletal alterations of the temporomandibular joint in the short term compared to untreated controls
Xinqi Huang et al. (2016)	China	The condylar position showed no changes after Herbst treatment. The condylar posterior space after Twin-block treatment averagely increased by 0.31 mm (P < 0.00001), whereas the condylar anterior space averagely reduced by 0.32 mm (P < 0.00001). Twin-block appliance enables forward movement of the condylar position

### Temporommandibular joint symptoms

Few cases in the included studies reported temporomandibular joint symptoms (32 of 962, 3.3%) during treatment time, but these temporary symptoms disappeared during follow-up time. The reported symptoms included TMJ noises, TMJ pain and oral facial pain, but no TMJ dysfunction.

### Condyle and glenoid fossa remodeling

In the studies of Popowich K [36], Ivorra-Carbonell L [37], Santana LG [9] and Kyburz KS [38], treated samples with permanent teeth were asked to take MRI before and after the removal of appliance to measure the mophology of TMJ region. Compared with pretreatment condition, both condyle and glenoid fossa remodeling was visually inspected through MRI, also an area of increased signal intensity in the posterior-superior region of the condyle was reported in the TMJs. Only 17 cases (1.8%) reported “osteoarthritic changes or deviations in condyle form” after treatment, but none of the samples reported TMJ dysfunction, either.

### Condylar and TMJ disc position

Xinqi Huang [39] stated in their study that after the treatment of twin-block appliance, the condylar posterior space increased whereas the condylar anterior space reduced. But they revealed that no significant change of both anterior and posterior space of condylar was observed after the treatment of Herbst appliance. Otherwise, Kurt Popowich [29], Santana LG [9] and Kyburz KS [38] all measured TMJ disc on MRI, they found that at the end of treatment, totally 47 patients (4.9%) had disc displacement, but during the follow-up year for about 1 year, the disc all had normal position. To conclude, functional mandibular advancement enables forward movement of the condylar position, but after the remodeling of glenoid and condylar, the TMJ disc will eventually have normal position without dysfunction of TMJ.

### Subgroup analysis

**Fig. 6:**
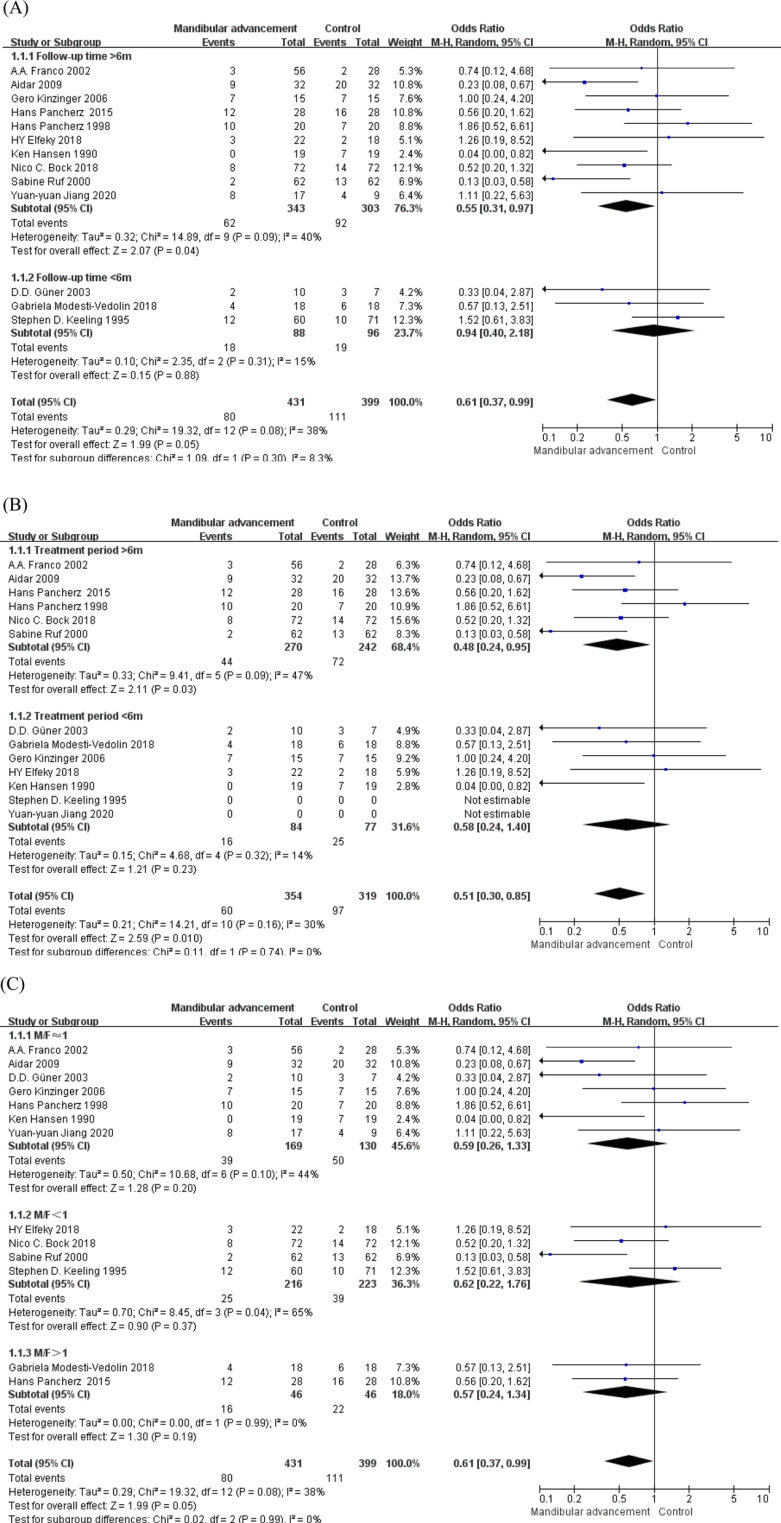

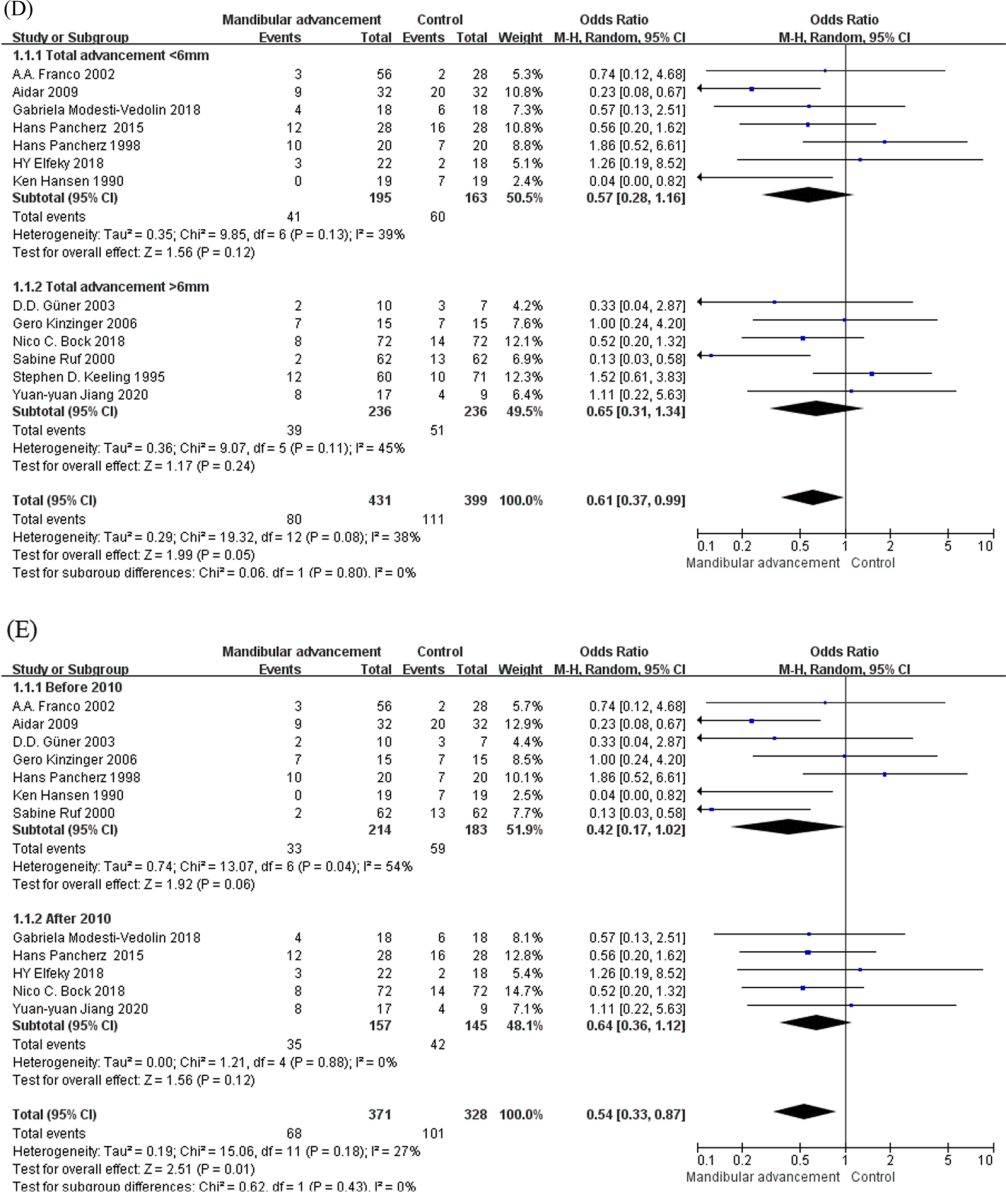
The result of subgroup analysis (**a**–**e**)

Demographic data were obtained, the included researches were mainly divided into 5 subgroups to find out the resource of heterogeneity according to follow-up year (over or under 1 year), treatment time (over or under 0.5 year), gender ratio (M/F > 1, M/F < 1 or M/F≈1), total amount of advancement (less or more than 6 mm) and publish year (before or after 2010), the result of subgroup analyses was performed in Fig. 6. The result suggested that subgroup 1 (follow-up year) had little influence on the generation of the heterogeneity. On the contrary, after subgroup analysis, I^2^ in subgroup 2–5 had decreased to 0%, which illustrated that these factors (gender ratio, treatment time, total amount of advancement and publish year)could be regarded as the chief resources of heterogeneity in this study. Further demonstration would be required in discussion.

## Discussion

### Summary of evidence

For this systematic review and meta-analysis, we evaluated the correlation between TMD and functional mandibular advancement. Following thorough database searches, it was observed that available researches were rare to obtain. Finally, 18 literatures were included in this systematic review. The quality of selected studies was able to answer this clinical question submitted in this systematic review. According to the criteria of grading evidence given by Cochrane Handbook, our study included 5 systematic reviews, 5 RCTs and 8 NRSIs. After quantitative assessment of meta-analysis, our study indicated that functional mandibular advancement would not cause TMD (P < 0.05).

Most of researches showed that after treatment, position of the condyle was more forward and glenoid fossa also remodeled, even in the patients who have anterior disc displacement with reduction (ADDR) [40], treatment-induced deterioration was not found in the disc-condyle relationship compared with pre-treatment condition, while the relationship was improved in some cases. The research published by Payam Owtad et al. [41] found that mandibular advancement could increase FGF8 factor in the TMJ region, which might promote adaptive remodeling of the TMJ disc, glenoid fossa and condyle, but it would cost a relatively long time to finish. Published researches had also reported some biochemical effects of functional mandibular advancement devices on the TMJ, even if the condyle was migrated to a more anterior position, no considerable stress was investigated on the TMJ structure [11, 29, 41, 42], but there was increasing pressure detected in masticatory muscle [43, 44]. Many researchers also found that adolescence appeared to have more active reconstruction in the TMJ region than adults [45, 46], so this might indicate that muscle pain and TMJ discomfort could release in a relatively short period in adolescent patients, especially in who still had growing potential.

As stated before in subgroup analyses, some other factors besides the intervention method could also affect the result. Follow-up time after treatment was regarded as one of the main factors. After categorization of the included case reports according to follow-up period (short-term and long-term), seven pieces of research set over 1-year follow-up in their experiment, and the outcomes showed that these patients’ TMJ morphology were as normal individuals, as well as no adverse effects were observed, also they didn’t show more tendency to develop TMD; the other pieces of research which observed patients just several months found that nearly 20% of the patients reported TMJ symptoms (including TMJ clicking and masticatory muscle pain). Many researchers [47, 48] reported patients who received such treatment were more likely to have their temporomandibular joint disc at a more forward position. Paula Loureiro Cheib et al.’s research [43] also indicated that immediately after functional appliance, the condyles were displaced anteriorly and inferiorly. The systematic review published by Kurt Popowich [29] also offered high-quality evidence to support the idea that although few patients had their TMJ disc position changed, such changes might cause TMJ discomfort momentarily. But after a period for TMJ to adapt, subjective symptoms and clinical signs seemed to disappear, and no obvious dysfunction was observed in TMJ. TMD is a type of self-limiting disease; existing research had also already reported that usually after 12 weeks of clinical observation, there was no discomfort complained by patients with disc displacement [21]. So on the bias of different follow-up time in each research and the outcome indicators we set in this research (mainly subjective symptoms),the result might vary a lot. But no considerable stress was detected on the TMJ structure when the condyle was migrated to a more anterior position overall [22, 44, 49], even in the patients complained about TMJ symptoms. Since TMJ discomfort after mandibular advancement devices wearing might be related to muscle dysfunction rather than pressure on TMJ itself [2, 27], and the function of masticatory muscle would recovery after a period of time, although bias and heterogeneity existed in this research, the overall statistical result still showed that there was no adverse side effect on TMJs after functional mandibular advancement.

### The correlation between gender and TMD

Gender can also be considered as a major factor that might affect the result. The studies published by Sabine Ruf [50], Niko C. Bock [51], HY Elfeky and Ken Hansen [42] all had bias on selecting samples, especially Ken Hansen’s study, their samples were all males and the sample HY Elfeky selected were all female. As Bueno CH et al. [53] stated in their systematic review that compared to men, the risk that women developed TMD was two times greater. So did Sójka A et al.’s observational research [54] on medical students in found that about one-third of the students in this study presented symptoms of TMD, and female students appeared to have a higher level of these symptoms. Tae-Yoon Kim et al. [55] gave evidence in their research that there may be physiological and pathological gender differences in TMD. There were more males in included samples than females in our study, since it already has evidence that women are more likely to have TMD, it might attribute to a bias in the final result.

### The correlation between total amount of advancement and TMD

Furthermore, to assess the relevant influence that total amount of advancement had on TMJs, we compared patients who received over or less than 6 mm total advancement and designed a subgroup. It had statistical significance in our research that over 6 mm advancement would not cause TMD after treatment. Knappe SW [56] and his colleagues compared these two groups in their study, during a long period of follow-up for 2 years, they observed a great amount of total mandibular advancement would not make patients have higher a risk to cause TMDs.

### The correlation between treatment period and having TMJ subjective symptoms

We also found that treatment time for functional appliance use would affect the outcomes, too. More patients underwent over 0.5 year treatment period complaining about TMJ symptoms than those who underwent treatment period less than 0.5 year. During treatment, the patient’s mandible was positioned more forward, and the condylar also migrated anteriorly, too [22]. Stress on the temporomandibular joint structures considerably increased, so it might lead to TMJ symptoms report. As the treatment time prolonged, minor functional disturbances in the masticatory system appeared [6, 57]. But these disturbances were temporary [58], the symptoms would finally disappear when the remodeling of condylar finished.

### The effect that existing TMD having on outcomes

Besides, patients with or without TMD before treatment will also affect the result. Peltola J S [27] took radiographic examination during follow-up time, they found that the condylar’s structure of patients with existing TMD had remained constantly. It suggested that the subjective symptoms and clinical signs seemed to cause the subjects no or only minor problems. Among all the included studies, only studies published by Sabine Ruf et al. [50] and Laura Ivorra-Carbonell et al. [30] concerned about both healthy patients and patients with pre-existing disorders, and their designed samples included these two types of patients. And they all found that functional mandibular advancement didn’t affect the normal structure of both condylar and glenoid fossa, along with condyle-glenoid fossa relationship in long-term. Laura Ivorra-Carbonell [30] summarized in their systematic review that after treatment, the position of the condylar was more forward, the glenoid fossa also remodeled and the adaption of condyle’s morphology was observed, no significant adverse effect on the TMJ was observed. Sabine Ruf [50] also pointed out in their study that total disc displacement with reduction or without reduction could not be regarded as contraindications of applying functional mandibular advancement. But the researchers also indicated that for high-risk patients (patients with pathological changes in condyle), the use of such treatment should be of careful attention [6, 59].

## Limitations

Limited high-quality RCTs were included in this study, which might lead to an inherent risk of bias. In addition, most articles didn’t provide us large samples to examine, and short-time observation may also cause bias. Also too many male patients included might cause bias on results, too. More high-quality studies with low bias are recommended.

## Conclusions

This study formulated a strict inclusion criterion to make the program of gathering and analyzing data more repeatable. The result of this meta-analysis performs that (1) TMJ symptoms may occur during the functional oral appliance wearing, but the symptoms will release or disappear during the follow-up period. (2) Less convincing evidence indicates that slightly pre-existing TMD will be improved after treatment. (3) There is TMJ disc anterior displacement observed during treatment, but most of them will return to the normal position later due to the remodeling of TMJ. (4) Moderate evidence support that FMA will not induce or aggravate TMD. But gender, follow-up year, the total amount of advancement, and treatment period may affect the result to a certain extent, so a more detailed and rigorous experiment should be designed to decrease the bias from gender, follow-up year, the total amount of advancement and treatment period.

## Supplementary Information

**Additional file 1:** Searching strategies.

**Additional file 2:** PRISMA flow diagram.

## Data Availability

The results of data extraction in this study are available from the corresponding author on reasonable request.
