# Supplementary material for: The effect of functional mandibular advancement for adolescent patients with skeletal class II malocclusion on the TMJ: a systematic review and meta-analysis
Source: BMC Oral Health. 2022 Mar 3;22:51. doi: 10.1186/s12903-022-02075-8 (PMC8895665; doi:10.1186/s12903-022-02075-8)
Supplement: Supplementary file 1 — Additional file 1: Searching strategies. [file 12903_2022_2075_MOESM1_ESM.doc]

Additional file 1 Searching strategies

| **PubMed** | |
| --- | --- |
| #1 | "Functional mandibular advancement"[Mesh] |
| #2 | ((((((((((Functional mandibular advancement[Title/Abstract]) OR Temporomandibular joint disease[Title/Abstract]) OR Temporomandibular joint[Title/Abstract]) OR Temporomandibular disorder[Title/Abstract]) OR Cranialfacial pain[Title/Abstract]) OR Condylar resorption[Title/Abstract]) OR Orthodontics[Title/Abstract] OR mandibular forward positioning[Title/Abstract] OR mandibular advancement [Title/Abstract] OR Herbst appliance[Title/Abstract] OR activator appliance[Title/Abstract] OR bionator appliance[Title/Abstract] OR twin-block appliance[Title/Abstract] OR Fränkel appliance[Title/Abstract] OR Forsus appliance[Title/Abstract] OR Class Ⅱ malocclusion [Title/Abstract]))) |
| #3 | #1 OR #2 |
| #4 | "Mandibular Advancement"[Mesh] |
| #5 | ((((((((((((((((((Mandibular forward positioning[Title/Abstract]) OR (Mandibular forward positioning[Title/Abstract]) OR(Cranialfacial pain[Title/Abstract]) OR Condylar resorption[Title/Abstract]) OR Orthodontics[Title/Abstract]) OR TMJ[Title/Abstract]) OR Class Ⅱ malocclusion[Title/Abstract]) |
| #6 | #4 OR #5 |
| #7 | "Randomized Controlled Trials"[Mesh] |
| #8 | ((((((Randomized Controlled Trial[Publication Type]) OR controlled clinical trial[Publication Type]) OR randomized[Title/Abstract]) OR placebo[Title/Abstract]) OR  randomly[Title/Abstract]) OR trial[Title/Abstract]) OR groups[Title/Abstract] OR Double-blinded method[Title/Abstract] OR Single-blinded method[Title/Abstract] |
| #9 | #7 OR #8 |
| #10 | #3 AND #6 AND #9 |
| **Embase** | |
| #1 | '(mandibular advancement'/exp |
| #2 | 'mandibular advancement':ab,ti OR 'temporomandibular joint disorders':ab,ti OR 'temporomandibular joint disease':ab,ti OR 'temporomandibular joint diseases':ab,ti OR  'cranialfacial pain':ab,ti OR 'condylar resorption':ab,ti OR 'orthodontics':ab,ti OR 'class Ⅱ malocclusion':ab,ti OR 'tmj diseases':ab,ti OR 'temporomandibular joint dysfunction syndrome':ab,ti OR 'functional mandibular advancement':ab,ti OR 'Herbst appliance':ab,ti OR 'activator appliance':ab,ti OR 'bionator appliance':ab,ti OR  'twin-block appliance':ab,ti OR Fränkel appliance':ab,ti OR 'Forsus appliance':ab,ti |
| #3 | #1 OR #2 |
| #4 | 'mandibular forward positioning'/exp |
| #5 | 'mandibular forward positioning':ab,ti OR ' class Ⅱ malocclusion':ab,ti OR 'temporomandibular joint disorders':ab,ti OR tmds':ab,ti OR 'tmj':ab,ti OR'cranialfacial  pain':ab,ti OR'condylar resorption':ab,ti OR'orthodontics':ab,ti |
| #6 | #4 OR #5 |
| #7 | 'randomized controlled trial '/exp |
| #8 | 'randomized controlled trial':it OR 'controlled clinical trial':it OR 'randomized':ab,ti OR  'placebo':ab,ti OR 'randomly':ab,ti OR 'trial':ab,ti OR 'groups':ab,ti OR 'double-blinded method':ab,ti OR 'single-blinded method':ab,ti |
| #9 | #7 OR #8 |
| #10 | #3 AND #6 AND #9 |

| **Cochrane** | | |
| --- | --- | --- |
| #1 | | MeSH descriptor: [functional mandibular advancement] explode all trees |
| #2 | | (functional mandibular advancment) OR temporomandibular joint disease) OR temporomandibular disorder) OR TMJ disorder) OR TMJ disease) OR temporomandibular joint dysfunction syndrome)OR cranialfacial pain) OR condylar resorption) OR orthodontics) OR mandibular forward positioning) OR mandibular advancement) OR Herbst appliance) OR activator appliance) OR bionator appliance) OR twin-block appliance) OR Fränkel appliance) OR Forsus appliance) OR Class Ⅱ malocclusion):ti,ab,kw |
| #3 | | #1 OR #2 |
| #4 | | MeSH descriptor: [randomized controlled trials] explode all trees |
| #5 | | (randomized controlled trial):pt OR (controlled clinical trial):pt OR (randomized) OR placebo) OR randomly) OR trial) OR groups) OR doubled-blinded method) OR single-blinded method):ti,ab,kw OR placebo):ti,ab,kw |
| #6 | | #4 OR #5 |
| #7 | | #3 AND #6 |
| **Web of Science** | | |
| #1 | (TS=(Temporomandibular Joint Disorders) OR TS=(Temporomandibular Joint Disorder) OR TS=(TMJ Disorders) OR TS=(TMJ Disorder) OR TS=(Temporomandibular Disorders) OR TS=(Temporomandibular Disorder) OR TS=(Temporomandibular Joint Disease) OR TS=(Temporomandibular Joint Diseases) OR TS=(TMJ Diseases) OR TS=(TMJ Disease) OR TS=(Temporomandibular Joint Dysfunction Syndrome)) | |
| #2 | (TS=(functional mandibular advancment) OR TS=(temporomandibular joint disease) OR TS=(temporomandibular disorder) OR TS=(TMJ disorder) OR TS=(TMJ disease) OR TS=(temporomandibular joint dysfunction syndrome)OR TS=(cranialfacial pain) OR TS=(condylar resorption) OR TS=(orthodontics) OR TS=(mandibular forward positioning) OR TS=(mandibular advancement) OR TS=(Herbst appliance) OR TS=(activator appliance) OR TS=(bionator appliance) OR TS=(twin-block appliance) OR TS=(Fränkel appliance) OR TS=(Forsus appliance) OR TS=(class Ⅱ malocclusion)) | |
| #3 | #1 OR #2 | |
| #4 | (TS=(random)) | |
| #5 | (TS=(randomized controlled trail) OR TS=(controlled clinical trail) OR TS=(randomized) OR TS=(trail) OR TS=(groups) OR TS=(double-blinded method)OR TS=(single-blind method) OR TS=(placebo) | |
| #6 | #4 OR #5 | |
| #7 | #3 AND #6 | |
| **Scopus** | | |
| #1 | INDEXTERMS( mandibular advancement) | |
| #2 | TITLE-ABS-KEY( mandibular advancement) OR TITLE-ABS-KEY( temporomandibular joint) OR TITLE-ABS-KEY( TMJ disorders) OR TITLE-ABS-KEY( TMJ disease) OR TITLE-ABS-KEY( TMJ disorder) OR TITLE-ABS-KEY( TMJ dysfunction syndrome) OR TITLE-ABS-KEY( condylar resorption) OR TITLE-ABS-KEY( orthodontics) OR TITLE-ABS-KEY( functional mandibular advancement) OR TITLE-ABS-KEY( forward mandibular repositioning) OR TITLE-ABS-KEY( Herbst appliance) OR TITLE-ABS-KEY( twin-block appliance) OR TITLE-ABS-KEY( activator appliance) OR TITLE-ABS-KEY( bionator appliance) OR TITLE-ABS-KEY( Fränkel appliance) OR TITLE-ABS-KEY( Forsus appliance) OR TITLE-ABS-KEY( class Ⅱ malocclusion) | |
| #3 | #1 OR #2 | |
| #4 | INDEXTERMS( randomized controlled trail) | |
| #5 | TITLE-ABS-KEY( randomized controlled trail) OR TITLE-ABS-KEY( controlled clinical trail) OR TITLE-ABS-KEY( randomized) OR TITLE-ABS-KEY( trail) OR TITLE-ABS-KEY( placebo) OR TITLE-ABS-KEY( groups) OR TITLE-ABS-KEY( single-blinded method) OR TITLE-ABS-KEY( double-blinded method) | |
| #6 | #4 OR #5 | |
| #7 | #3 AND #6 | |
