# Supplementary material for: The effect of functional mandibular advancement for adolescent patients with skeletal class II malocclusion on the TMJ: a systematic review and meta-analysis
Source: BMC Oral Health. 2022 Mar 3;22:51. doi: 10.1186/s12903-022-02075-8 (PMC8895665; doi:10.1186/s12903-022-02075-8)
Supplement: Supplementary file 2 — Additional file 2: PRISMA flow diagram. [file 12903_2022_2075_MOESM2_ESM.doc]

Additional file 2 PRISMA flow diagram

**Identification**

**Screening**

**Eligibility**

**Included**

Researches retrived from database searching

(n=1015)

Pubmed

n=278

EMBASE

n=269

Cochrane

n=83

WOS

n=222

Articles after removing duplicate reports

n=733

After titles and abstracts checking

n=48

Unrelated studies(n=327)

Case reports(n=25)

Excluded studies

n=333

After full-text assessing for eligibility

n=25

Studies pooled in quantitative assessment (meta-analysis)

n=13

N=5(systematic review)

Scopus

n=162

Excluded studies for their low quality

n=3

Excluded studies for reasons

n=4
